# Mangled Extremity Severity Score - is it really useful in assessing limb salvage following open and crush injuries in the lower extremity?

**DOI:** 10.1186/1865-1380-7-S1-P6

**Published:** 2014-07-25

**Authors:** Thomas Kurien Bhanu

**Affiliations:** 1 Christian Medical College, Vellore, India

## Objective

To assess the utility of Mangled Extremity Severity Score in the injured lower limb.

## Methods

This study was done in the Accident & Emergency Centre of a tertiary hospital in India.

This is a descriptive study assessing the validity of the MESS score in predicting limb salvage.

The study included patients older than 18 years with open injuries/crush injuries of the lower extremities up to the level of the ankle (Gustilo and Anderson type III B / III C injuries).

Patients were divided into those who presented within 6 hours and those after 6 hours of injury. The final study consisted of 50 patients with 51 lower limb injuries. The study was conducted over a span of 18 months.

## Results

• The MESS mean score in the salvaged group were 4.36 and in the amputated group were 8.42.

• Thus the sensitivity was 75%, specificity 92.31%, positive predictive value 75% and negative predictive value 92.31%.

• After calculation of the receiver operator characteristic (ROC) curves and calculation of area under the curves (AUC) as 0.935, a score of 7 or greater predicted amputation with the highest sensitivity and specificity.

## Limitations

As this is a prospective study in a limited time frame, the long term follow up is not known. It is also not known from our study nor from other previous studies whether the MESS distinguishes between functional and nonfunctional salvage.

## Conclusion

A total score of ≥7 is significantly associated with amputation both in the primary amputation group as well as in the combined primary and delayed amputation group.

Thus the utility of MESS as a tool in assessing limb salvage following open and crush injuries to the lower extremity has been validated.

